# Black Widow Spider Envenomation and Cardiovascular Complications

**DOI:** 10.7759/cureus.73342

**Published:** 2024-11-09

**Authors:** Parmjot Khakh, William Q Lavercombe, Juan M Farina, Meera Kapadia, Gabriel M Aisenberg, Adrian Baranchuk

**Affiliations:** 1 Internal Medicine, Queen's University, Kingston, CAN; 2 Internal Medicine, Vanderbilt University Medical Center, Nashville, USA; 3 Cardiology, Mayo Clinic, Phoenix, USA; 4 Internal Medicine, University of Texas at Houston, Houston, USA; 5 Internal Medicine, University of Texas Health Science Center at Houston, Houston, USA; 6 Cardiology, Queen's University, Kingston, CAN

**Keywords:** alpha-latrotoxin, black widow spider, cardiovascular disease, latrodectus, myocarditis

## Abstract

Envenomation (latrodectism) with black widow spider (BWS) venom can cause dysfunction in the cardiovascular system. The pathophysiology and consequences of cardiovascular effects have not been fully elucidated. This review followed the Preferred Reporting Items for Systematic Reviews and Meta-Analyses (PRISMA) guidelines. Of 364 initial results, 22 articles (20 case reports and two case series) were used with 25 patients in total. Seventeen (68%) patients had hypertension, and 16 (64%) had tachycardia. High troponin levels were found in 16 (64%) patients. Electrocardiographic changes consisted of nine (36%) patients with ST-T segment abnormalities and three (12%) patients with supraventricular tachyarrhythmias. Wall motion abnormalities were present in 11 (44%) patients, with three of the patients experiencing left ventricle global hypokinesis. A reduced ejection fraction was present in 10 (40%) patients. Only six (24%) patients received antivenom and were discharged with no further complications. In conclusion, raising awareness for cardiovascular complications could be useful as there are no pathognomonic features in imaging or electrocardiogram (ECG). The impact of antivenom on cardiovascular complications has not been prospectively studied.

## Introduction and background

Black widow spider (BWS) envenomation and its complications are relevant given its prevalence around the world, its potential severity, and the ability for a low-cost and accessible treatment option. In this paper, we aimed to review the available evidence regarding cardiovascular involvement in BWS envenomation.

**Figure 1:**
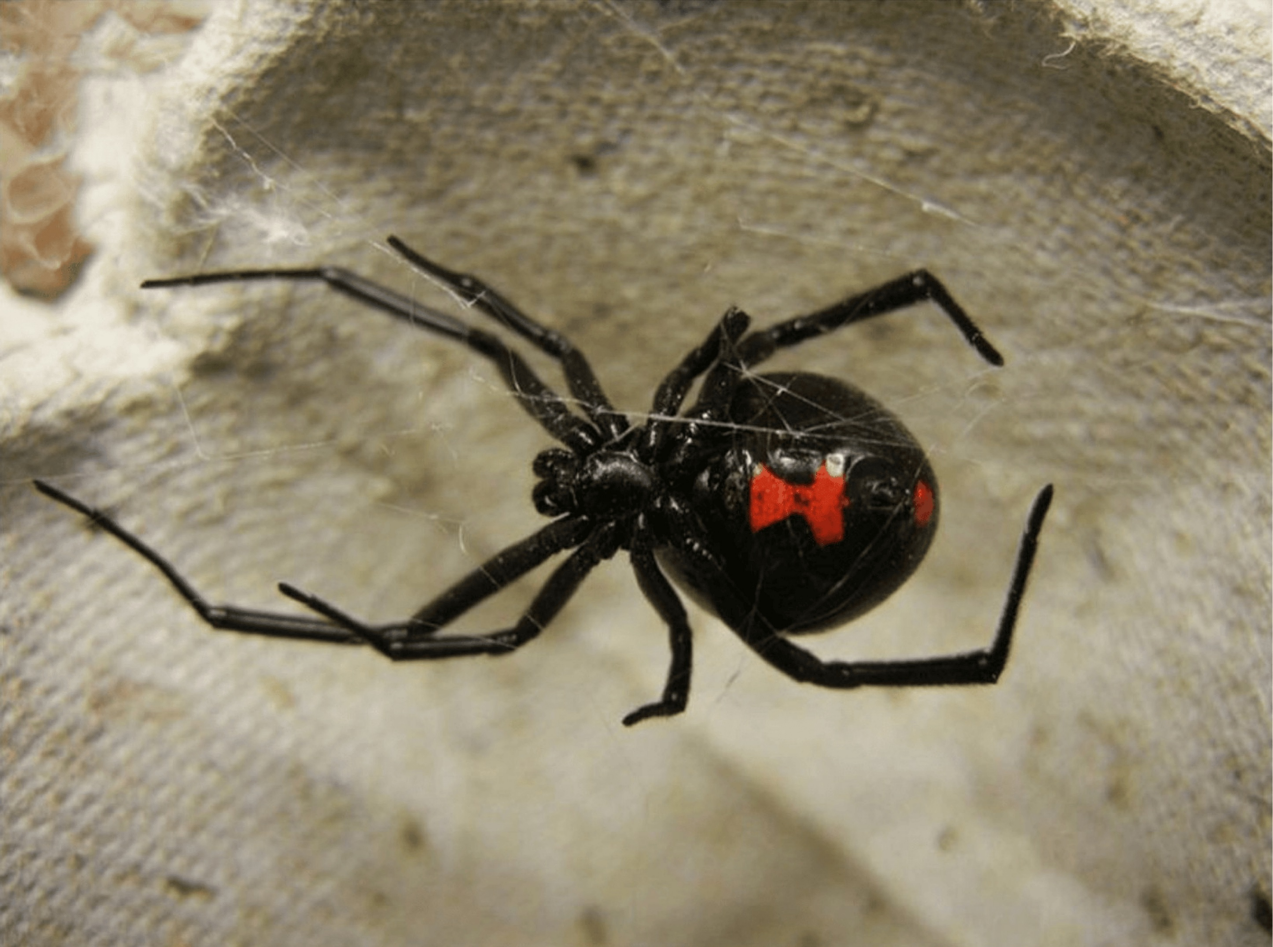
Appearance of a Latrodectus mactans specimen Image credit: Shenrich91 at https://commons.m.wikimedia.org/w/index.php?search=Black+widow+spider&title=Special:MediaSearch&type=image

The genus *Latrodectus* is found throughout the world, and five species are found in North America. *Latrodectus*, also known as the black widow spider, is recognized by its characteristic red, hourglass-shaped abdominal mark. They are 12-15 mm in size and can survive in a variety of climates. BWS have a characteristic mouthpiece (termed chelicerae), which makes them capable of delivering their venom through human skin, an adaptation not all spiders have (Figure 1) [1-7]. The female spider is more dangerous than the male because of longer fangs and larger venomous glands. Systemic symptoms and signs from *Latrodectus* spiders’ envenomation develop in approximately 33% of bitten patients. Of these bites, approximately 1%-2% progress to latrodectism, a potentially life-threatening condition requiring intensive care admission [4-8].

Symptoms are a consequence of the alpha-latrotoxin protein present in the spider venom. This toxin causes excessive release of acetylcholine and noradrenaline in the nervous system. Alpha-latrotoxin affects primarily the nervous system, although other tissues, such as the heart and lungs, are also targets of its toxic effect [4]. Older series describe uncommon cardiac complications, although potentially fatal if detection and treatment are not implemented early [9]. The pathophysiology of the cardiovascular impact is yet to be fully elucidated, in part because of its low frequency and in part because the world regions where the incidence of latrodectism is higher produce scarce literature on the topic.

## Review

Methods

We conducted a systematic search in the MEDLINE/PubMed database to select publications detailing the cardiovascular effect of BWS. Article selection was based on the following inclusion criteria: (a) publications issued until December 2023, (b) human studies, (c) articles relating to cardiovascular involvement in BWS envenomation, (d) written in any language as long as a translation into English was available (including Google’s automatic translation system), and (e) case reports and case series, clinical trials, prospective studies, retrospective studies, and guidelines. When the connection between BWS and cardiovascular involvement could not be confirmed, articles were excluded.

The following MeSH terms were used: “black widow”, “black widow spider”, “black widow envenomation”, “Latrodectus”, “heart”, “heart diseases”, “arrhythmias”, “myocarditis”, “pericarditis”, and “endocarditis”. Articles identified manually searching the references of included studies were added.

Two investigators (PK and WL) reviewed the titles and abstracts of the retrieved articles independently. All authors reviewed the studies independently to ensure accuracy and consistency and address the risk of bias. Disagreements were resolved by consensus. This review process followed the Preferred Reporting Items for Systematic Reviews and Meta-Analyses (PRISMA) guidelines. The goal of this article is to synthesize knowledge about cardiovascular involvement in black widow bites, its diagnosis, and management.

We extracted and methodically tabulated the following information from each of the chosen articles: the country of origin, the patient’s age and sex, vital signs, serum troponin levels, electrocardiogram and echocardiogram results, the patient’s outcomes, and whether or not antivenom therapy was administered.

Results

**Figure 2:**
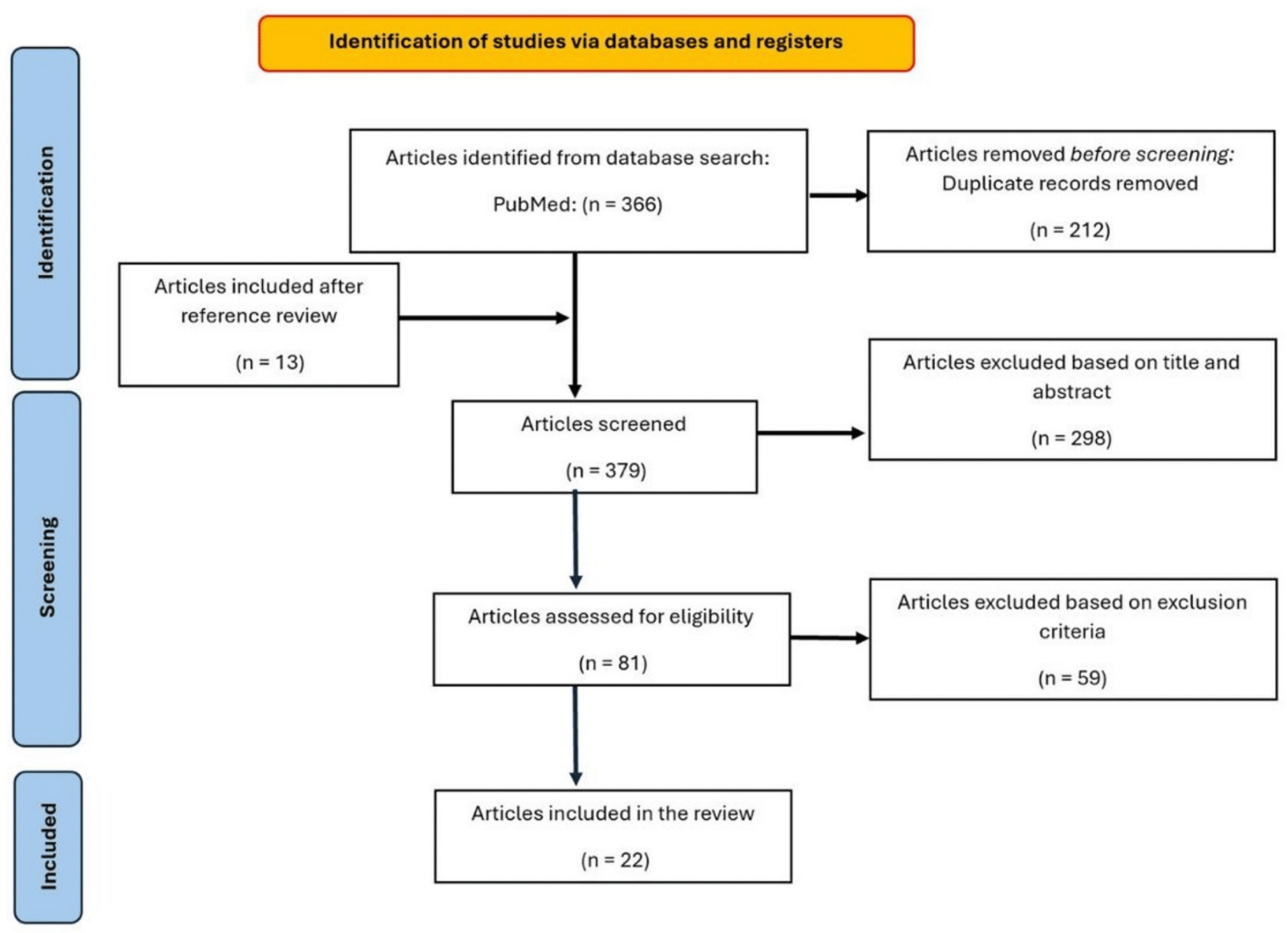
PRISMA flow diagram PRISMA: Preferred Reporting Items for Systematic Reviews and Meta-Analyses

The initial electronic search found 364 results. After screening titles and abstracts, 81 articles met our eligibility criteria, of which 22 articles were considered relevant and included in this systematic review. The selection consisted of 20 case reports [1-6,10-23] and two case series [24,25]. Figure 2 displays the PRISMA flow diagram. Table 1 summarizes each study discussing cardiac manifestations and clinical outcomes in patients with black widow envenomation. We did not tabulate the results from a case series where the individual manifestations and workup were not described; however, we presented some of their data [26].

**Table 1:** Summary of available publications on black widow spider envenomation with cardiac involvement ECG: electrocardiogram, CV: cardiovascular, EF: ejection fraction, HF: heart failure, TTE: transthoracic echocardiogram, LV: left ventricle, CK-MB: creatine kinase-MB, BNP: B-type natriuretic peptide, PVC: premature ventricular contraction, TAPSE: tricuspid annular plane systolic excursion, ED: emergency department

Reference	Age	Gender	Country of origin	Cardiovascular effects	Cardiac enzyme values	ECG	Echocardiogram	CV outcomes	Received antivenom
[1]	15	Male	Turkey	160/100 mmHg, pulse: 104 bpm	Troponin I: 1.4 ng/mL	ST-segment elevation in leads DI and aVL, and ST-segment depression in leads DII, III, aVF, and V4-V6	EF 30%, global hypokinesis of left ventricle	Patient treated for HF and was back at baseline within 48 hours. TTE obtained four weeks later revealed normal LV size with recovery of systolic function (EF: 60%).	No
[2]	22	Male	Turkey	200/120 mmHg, pulse: 77 bpm	Troponin: 0.63 ng/mL, CK-MB: 26.1 IU/L	Atrial depolarization abnormalities in leads DII, III, and aVF, and depolarization abnormalities in leads V1 and aVL	EF: 50%, anterior and septal wall motion abnormality	Patient recovered within 7 days.	No
[3]	19	Female	Greece	100/45 mmHg, pulse: 125 bpm; 2 hours later: 75/50 mmHg, pulse: 180 bpm	None	Rapid atrial fibrillation with incomplete right bundle branch block	EF less than 20%, dilatation of the four cardiac chambers, and severe global hypokinesis of the left ventricular wall	Patient died 36 hours after the bite.	No
[4]	26	Male	USA	162/100 mmHg, pulse: 109 bpm	Troponin: normal, BNP: 500 pg/mL	Sinus tachycardia	EF: 50%-55% without wall motion abnormalities or pericardial effusion	Patient discharged on day 5.	Yes
[5]	65	Male	Turkey	115/75 mmHg, pulse: 101 bpm	Troponin I: 6.1 ng/mL	0.5 mm ST-segment elevation in leads II, aVF, and V3 through V6; augmentation in T-wave amplitude in leads V3 through V6 without reciprocal changes	Normal	CV symptoms resolved within 12 hours.	No
[6]	35	Male	Morocco	-	-	-	EF: 48%, hypokinesis of the middle segments of the posterior and inferior wall of the left ventricle	Patient discharged in stable condition.	No
[10]	64	Female	Greece	150/80 mmHg, pulse: 104 bpm (sinus)	Elevated troponin I, CK-MB (no values provided)	T-wave flattening in aVL lead	EF: 36%, increased left ventricular end-systolic diameter with end-diastolic diameter preserved, increased filling pressures, hypokinesis of the basal and middle segments of the left ventricular walls	Cardiac troponins and echocardiogram were normal by day 6.	No
[11]	12	Female	Turkey	132/89 mmHg, pulse: 86 bpm	Troponin: 0.145 ng/mL, BNP: 4577 pg/mL	Sinus rhythm with two unifocal PVCs	EF: 62%, mild dilation of the left ventricle, mild mitral valve regurgitation, and mild aortic valve regurgitation	Patient discharged with no complications.	No
[12]	22	Male	Turkey	155/88 mmHg, pulse: 110 bpm (sinus)	Troponin I: 0.235 ng/mL, CK-MB: 122 IU/L	Sinus tachycardia and nonspecific ST-T-wave changes	Normal	Patient discharged with no complications.	No
[13]	84	Male	USA	192/64 mmHg, pulse: 66 bpm	Troponin: 0.13 ng/mL, CK-MB: 4 IU/L	Sinus rhythm	EF: 60%, moderate pulmonary hypertension, and trivial pericardial effusion without evidence of pericarditis	Patient discharged on day 4.	No
[14]	22	Male	USA	157/94 mmHg, pulse: 103 bpm	Troponin: 1.37 ng/mL	Incomplete right bundle branch block, precordial ST elevations	EF: 35%-40%, mild to moderate tricuspid regurgitation, and right ventricular systolic pressure of 47 mmHg	Patient discharged without complications.	Yes
[15]	15	Male	Turkey	160/100 mmHg, pulse: 124 bpm	Troponin I: 1.8 ng/mL	ST-segment depression at II, III, aVF, I, aVL, and V3-V6	EF: 22%, mildly dilated LV with global hypokinesis, grade 1 diastolic dysfunction, mitral regurgitation, pulmonary artery systolic pressure 35 mmHg, normal right ventricular dimensions, 16 cm of TAPSE, and pericardial fluid in the adjacency of the right ventricle	By day 6, echocardiography showed normal LV dimensions with EF of 62%, normal diastolic function, mild mitral regurgitation, and no pericardial effusion.	No
[16]	35	Male	Morocco	221/130 mmHg, pulse: 120 bpm	Troponin I: 1.93 ng/mL	Sinus tachycardia, increase of T-wave amplitude in leads V3and V4 with 3 mm ST-segment elevation	EF: 48%, midventricular septum, anterolateral, and inferior wall hypokinesis, and moderate mitral regurgitation	Patient discharged on day 6. Two weeks after discharge, TTE showed improvement in LV function of 50%.	No
[17]	2	Female	USA	140-160/110 mmHg, tachycardic	-	-	-	After 2 days on nifedipine, blood pressure was controlled, and the patient was discharged.	No
[18]	50	Male	Italy	-	Troponin I: 4.323 ng/mL, BNP: 1995 pg/mL	Diphasic T-waves in lateral leads	EF: 48%, wall motion of the LV, moderate systolic dysfunction (hypokinesia of the LV middle/basal segments of the inferior, lateral and inferior-lateral walls)	Patient discharged on day 11 with repeat TTE showing an EF of 55% and normalized cardiac enzymes.	No
[19]	35	Male	Egypt	150/100 mmHg, pulse: 110 bpm	Troponin I: 5.1 ng/mL, CK-MB: 89.9 IU/L	0.5 mm ST-segment elevation in leads I, and aVL with reciprocal ST-segment depression in leads II, III, aVF, and V2-V6	EF: 42%, regional wall motion abnormalities including hypokinesis of the mid-basal anterior, mid-basal postero-septal, mid-lateral, and basal inferior walls, with preserved thickness	Patient discharged on day 6 with resolution of symptoms, normalized ECG, and EF of 51%.	No
[20]	40	Male	Greece	Normal blood pressure and pulse	Troponin I: 0.9 ng/mL	Normal	Normal	Patient discharged without complications.	No
[21]	42	Female	France	170/110 mm Hg, pulse: 110 bpm (sinus)	Troponin I: 7.93 ng/mL	Normal	EF: 30%, anteroseptal akinesis and severe apical and anterolateral hypokinesis	Patient discharged on day 7 with normal troponin I. Follow-up TTE 15 days later showed an EF of 64% with LV hypertrophy.	No
[22]	36	Female	USA	156/81 mmHg, pulse: 92 bpm	-	-	-	Pregnant patient discharged after 1 day.	Yes
[23]	25	Male	Israel	240/110 mmHg, atrial fibrillation at 210 bpm	CK normal	Atrial fibrillation with rapid ventricular response	-	High levels of catecholamines noted on admission; discharged after 6 days with normal ECG and CK levels.	No
[24]	20	Male	Turkey	80/60 mmHg, pulse: 110 bpm	Troponin: 14.44 ng/mL	Sinus rhythm	Minimal LV wall movement disorders	Patient discharged on day 5.	No
[24]	33	Male	Turkey	135/90 mmHg, pulse: 84 bpm	Troponin: 6.26 ng/mL	Sinus rhythm	Normal	Patient discharged on day 3.	No
[25]	3	Male	USA	111/68 mmHg, pulse: 103 bpm	-	-	-	Patient discharged after day 3 in the hospital.	No
[25]	35	Male	USA	146/72 mmHg, pulse: 71 bpm	-	-	-	Patient discharged after administration of antivenom in the ED.	Yes
[25]	24	Female	USA	143/94 mmHg, pulse: 101 bpm	-	-	-	Patient experienced significant relief of pain after antivenom and was discharged from the ED.	Yes
[25]	8	Male	USA	134/92 mmHg, pulse: 96 bpm	-	-	-	Patient experienced significant relief of pain after antivenom and was discharged from the ED.	Yes

Latrodectus Species

The genus *Latrodectus* includes 30 species worldwide. Only five species are found in North America. They typically thrive in hot environments and exist on all continents except for Antarctica. The southern black widow spider (*Latrodectus mactans*) is found primarily in the southern United States [25]. The northern black widow (*Latrodectus variolus*) can be seen from eastern Texas to the East Coast. The western black widow (*Latrodectus hesperus*) is found in abundance in the southwestern United States and extends to southern British Columbia, southern Saskatchewan, and Alberta. The red widow (*Latrodectus bishopi*) and the brown widow (*Latrodectus geometricus*) are found predominantly in Florida [13,17]. Each year, the National Poison Data System (NPDS) receives approximately 2,600 reports on *Latrodectus* species exposures in the United States [27], whereas in a review of more than 10 million visits to the emergency department due to non-canine bites and stings in a 10-year span from 2001 to 2010, the authors reported 46,999 bites due to this spider genus [28].

Symptoms and Signs

**Figure 3:**
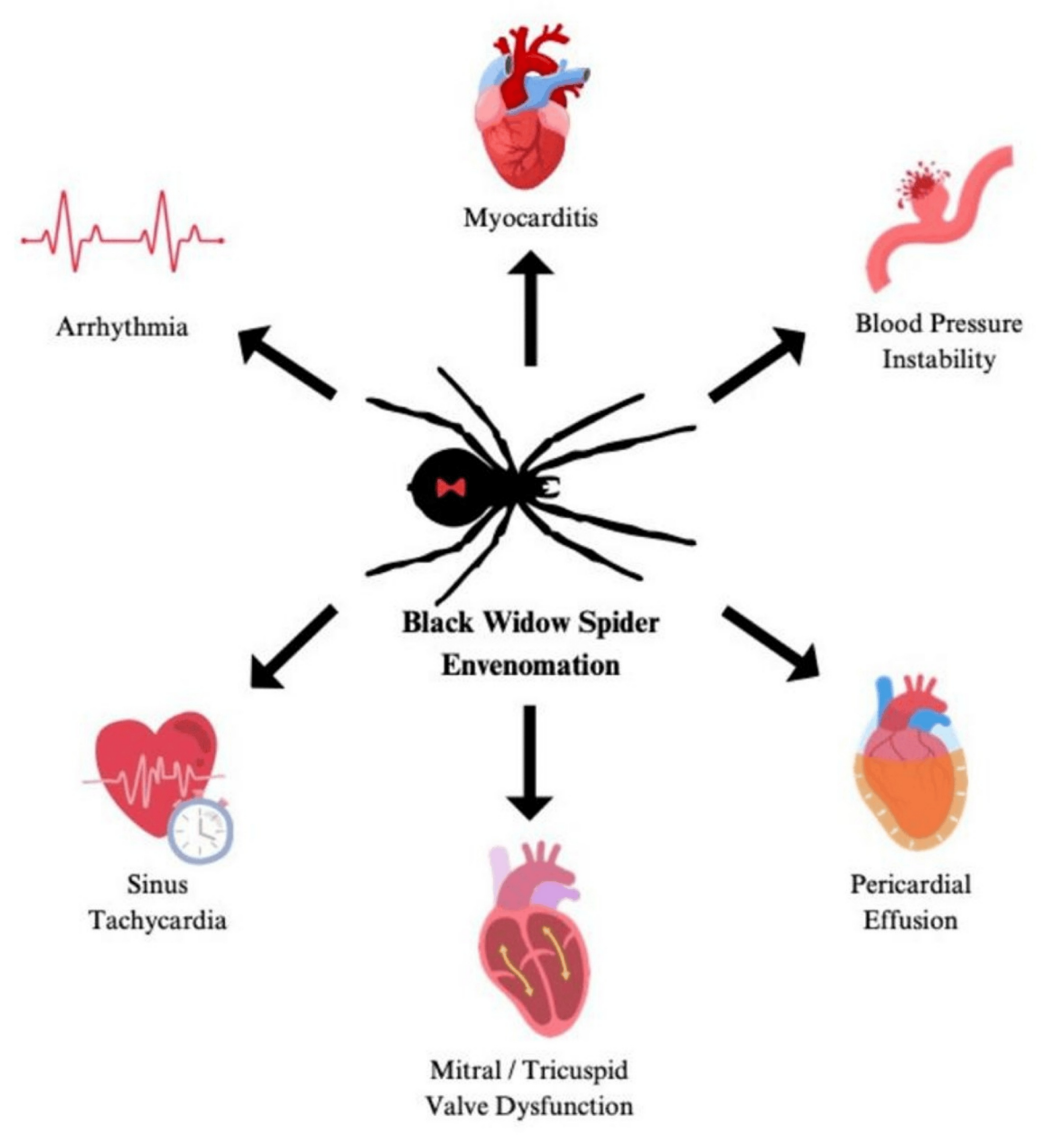
Infographic summarizing the cardiovascular manifestations attributed to black widow spider envenomation Image credit: William Lavercombe

Figure 3 summarizes the key symptoms. After *Latrodectus* bites, initial symptoms include a slight paresthesia eventually followed by increasing pain at the site of the bite during the first hour and involvement of the inguinal or axillary region within 5-15 minutes as the venom spreads through the lymphatic system. These symptoms are a consequence of both local inflammatory responses and the alpha-latrotoxin protein in the spider venom. Patients may experience a wide variety of local and systemic symptoms following BWS envenomation. Local presentations at the puncture site commonly include radiating and intense pain, edema, ecchymosis, and erythema.

Common systemic symptoms include severe pain, stiffening and cramps in the abdomen, cold perspiration, hypertension, tachycardia, piloerection, nausea, vomiting, malaise, and dyspnea [1-7]. Patients can develop cardiovascular complications, including arrhythmias, chest pain, and ventricular dysfunction with pulmonary edema and hypoxia. Seventeen patients had hypertension, and 16 were tachycardic. In a cohort including 163 patients, 45 patients had high blood pressure (only six of them had a reported history of hypertension), and 14 had tachycardia. Twenty-five patients reported isolated chest pain [26].

High troponin levels were found in 16 patients. In a series, seven (4%) of 163 patients had elevated creatine kinase serum levels [26]. In that study, only four electrocardiograms were completed with only one reported as abnormal. Electrocardiographic changes otherwise consisted of nine patients with ST-T segment abnormalities and three patients with supraventricular tachyarrhythmias. Wall motion abnormalities were present in 11 patients with three of the patients experiencing left ventricle global hypokinesis. A reduced ejection fraction was present in 10 patients. Only five patients received antivenom and were discharged with no further complications. Unlike older casuistic [9], we did not find reports of fatal cases in the literature.

Pathophysiology

*Latrodectus* venom is one of the most potent poisons by volume. The primary toxin responsible for the envenomation syndrome in humans is alpha-latrotoxin, a 120 kilodalton protein, which binds to the presynaptic neuron and results in the exocytosis of multiple neurotransmitters, including norepinephrine, acetylcholine, and glutamate from the presynaptic cell [23]. Alpha-latrotoxin can bind to the presynaptic receptor known as l-atrophin (also called the calcium-independent receptor for alpha-latrotoxin). The exact series of events after the toxin binds its receptor is not known but may involve either phospholipase C signaling with IP3-induced mobilization of intracellular calcium [11,13], or it might involve a different and unknown G protein/second messenger system. Any of these results in the mobilization of intracellular calcium and subsequent exocytosis of neurotransmitters even in the absence of extracellular calcium [4,14].

The presence of blood pressure variation is likely secondary to the initial catecholamine surge associated with alpha-latrotoxin causing hypertension and then subsequent LV dysfunction resulting in reduced cardiac output and hypotension. The arrhythmias seen with alpha-latrotoxin are nonspecific and have not been associated with any ventricular dysrhythmias or atrioventricular (AV) dissociation.

The causes of specific electrophysiological changes as well as coronary and myocardial involvement are not clearly understood. The catecholamine surge produced by alpha-latrotoxin results in myocardial stunning, which is likely the mechanism of the development of rapidly reversible myocardial dysfunction and heart failure [14]. Elevated plasma catecholamine levels play a major role in the mechanism of stress-related myocardial stunning due to sudden emotional stress, as seen in patients with takotsubo cardiomyopathy [10]. Catecholamine release triggered by the direct effect of alpha-latrotoxin in catecholamine-secreting cells or catecholamines secreted because of the emotional and physical stress of the spider bite itself or a combination of both might have been the mechanism causing the transient cardiomyopathy in these patients [10].

Workup

There is no test that proves the spider bite but collecting the right history. It is noteworthy that most patients will not seek help after a spider bite. Therefore, these recommendations apply to patients who present with more serious pictures. At a minimum, all patients should have an electrocardiogram (ECG), basic blood work, and serum troponin levels. Troponin elevation does not necessarily correlate with the severity of symptoms, but elevation can be helpful in risk stratifying the severity of a patient’s presentation. Cardiovascular manifestations of BWS envenomation should be treated on a case-specific basis.

BWS envenomation can cause left ventricular dysfunction with myocardial stunning resulting in an increased risk of the development of decompensated heart failure. If clinical or patient factors indicate concerning symptoms, patients should undergo an urgent echocardiogram. ECGs should be closely assessed for other nonspecific yet previously described abnormalities (ventricular extrasystoles, prolonged QTc intervals, left bundle branch block, T-wave inversion, T-wave flattening, tall T-waves, and presence of U-waves) and compared to previous if possible.

The need for escalation in diagnostic tests is facility- and provider-dependent. Cardiac MRI and angiograms are not commonly needed, for they do not change the patient’s diagnosis or management [6]. Cardiac MRI may be used as an alternative to biopsy in the acute stage (in the first five days) for a patient presenting with symptoms suggesting myocarditis. It can be used to rule out myocardial ischemia from plaque rupture as it can show a T2 myocardial hypersignal, sub-epicardial focal uptake of contrast on delayed sequences after injection, without systematization within a vascular territory, and remains in the subendocardium [6].

Management

Immediately following a BWS bite, patients should be advised to keep the injured area immobilized while removing any restrictive clothing that may constrict the limbs in case swelling occurs. Patient vital signs should be monitored, supportive care administered as necessary, and intravenous access secured.

Telemetry can be helpful in monitoring the development of cardiac complications. Hypertensive crises are treated as in patients without BWS envenomation. Patients with myocarditis and decompensated heart failure benefit from high-level care and possible admission to intensive care units. Appropriate observation time before discharge is dependent on patient factors and the severity of initial symptoms. Most patients presenting with cardiac involvement stay in the hospital for a minimum of two days. If the patient presents without symptoms concerning systemic illness, most can be discharged the same day after ensuring a normal ECG, bloodwork, and hemodynamics. In this review, we found durations of admission going from hours to 11 days.

In the absence of better evidence, ECG and clinical follow-up should be scheduled after hospitalization in patients with cardiovascular consequences to screen for possible late-onset complications, as well as monitoring for antivenom-related serum sickness among patients who received that therapy.

The treatment of BWS envenomation depends on the severity. In turn, severity depends on whether the symptoms are local and related to the bite (grade 1), the pain extends beyond that area without causing changes in vital signs (grade 2), or there is generalized diaphoresis, headaches, nausea, vomiting, and changes on vital signs (grade 3). For most patients, the use of muscle relaxers and opioids is sufficient, although in a large series, the use of antivenom in patients with grade 2 and 3 severity was associated with a faster resolution of symptoms, a lower admission rate, and a slightly shorter length of in-hospital stay [26]. In our review, we did not find studies focused on the therapy of the cardiac involvement of BWS envenomation.

Therefore, the administration of BWS antivenom is decided on a case-by-case basis. Antivenom is a horse serum-derived product containing IgG antibodies. It is frequently used for symptoms refractory to opioids and benzodiazepines or life-threatening conditions, such as uncontrolled hypertensive emergencies or organ injury (including myocarditis), although it has been used in milder cases [13,22]. Antivenom is proven to control the histopathological changes resulting from experimental envenomation in animal models [29]. The antivenom is inexpensive and very rarely associated with allergic manifestations [30]; however, it is not available in every country, which can limit its administration. Discussing the patient and clinical situation with the local regulating body would be most beneficial to ensure timely retrieval and administration of it should it be required.

Antivenom binds latrotoxin and prevents its interaction with presynaptic membranes, therefore preventing the progression of the disease. Patients typically see improvement within 30-60 minutes. The administration of BWS antivenom was followed by fewer treatment failures than placebo treatment [7]. Among 9,872 cases of BWS envenomation, the decision to use antivenom was progressively more frequent as severity progressed. In this series, there were two deaths by cardiac arrest attributed to the envenomation, but not to side effects to the antivenom [31]. Considering these factors, it is of utmost importance that physicians in endemic areas be trained and equipped to provide these therapies and to refer cases that require more complex care. There are conflicting data on how antivenom should be administered, as one study found that there is no difference in the antivenom serum concentration after intravenous or intramuscular administration [32], while another study found that intravenous administration of antivenom permitted its detection in blood while intramuscular did not [33].

Discussion

To our knowledge, this is the first systematic review on BWS envenomation and cardiovascular involvement. BWS envenomation causing cardiovascular complications may be underestimated due to underreporting and diagnostic barriers. Low incidence, lack of information, and the high heterogeneity of cardiovascular manifestations can represent a diagnostic challenge for health teams. Current literature primarily stems from regions where the BWS is endemic and may not accurately reflect its prevalence given the lower threshold of suspicion for its diagnosis in other locations. Cardiovascular manifestations include different conditions with a wide spectrum of severity, ranging from mild cases to life-threatening situations. The potential severity of cardiovascular complications should be considered by healthcare teams in endemic areas to pay special attention to their rapid diagnosis and treatment.

Raising awareness of cardiac complications could be useful as there are no pathognomonic features in imaging or ECG. This has been helpful in other endemic diseases such as Chagas, where online modules were given and caused significant increases in knowledge about the pathogen [34].

Individuals in tropical and rural regions may have limited access to medical centers equipped with adequate amounts of antivenom and technology for associated supportive care and should have a low threshold for moving patients to primary care. It is known that access to antivenom is dependent on manufacturer availability and most importantly, geographical location.

The limitations of this systematic review include firstly the low number of available cases. With only 25 cases and the reported heterogeneity, it is difficult to derive strong recommendations when we encounter our next case. Secondly, the lack of data confirming the specific pathophysiology of cardiac involvement represents a barrier to a comprehensive therapeutic plan. Thirdly, the significant variability between patient populations and geographic locations may create a potential bias in terms of the severity of presentations and response to therapy when the subspecies of spiders vary, as well as underreporting in areas where awareness and diagnostic capacity are limited. Finally, the decision to administer antivenom did not follow a specific pattern.

## Conclusions

Black widow spider (BWS) envenomation and its complications are relevant given its global prevalence, its potential severity, and the ability for a low-cost and accessible treatment option. After our review, we conclude that the CV manifestations of BWS envenomation are heterogeneous and include chest pain, tachycardia, hypertension, and heart failure. Abnormal cardiac biomarkers can be detected. Mortality seems exceedingly rare.

The current knowledge on the impact, pathophysiology, clinical presentation, and management of the cardiovascular involvement in BWS envenomation is limited. An algorithmic approach to the use of antivenom, randomization of patients regarding the use of antivenom versus placebo, and the length and modality of post-envenomation care are all needed considerations and opportunities for further research.
